# Reinforced Hyaluronic Acid‐Based Matrices Promote 3D Neuronal Network Formation

**DOI:** 10.1002/adhm.202201826

**Published:** 2022-09-01

**Authors:** Dieter Janzen, Ezgi Bakirci, Jessica Faber, Mateo Andrade Mier, Julia Hauptstein, Arindam Pal, Leonard Forster, Jonas Hazur, Aldo R. Boccaccini, Rainer Detsch, Jörg Teßmar, Silvia Budday, Torsten Blunk, Paul D. Dalton, Carmen Villmann

**Affiliations:** ^1^ Institute for Clinical Neurobiology University Hospital Würzburg Versbacherstr. 5 97078 Würzburg Germany; ^2^ Department of Functional Materials in Medicine and Dentistry and Bavarian Polymer Institute University Hospital Würzburg Pleicherwall 2 97070 Würzburg Germany; ^3^ Department of Biomedical Engineering Carnegie Mellon University Pittsburgh PA 15213 USA; ^4^ Department of Mechanical Engineering Institute of Applied Mechanics Friedrich‐Alexander University of Erlangen‐Nürnberg Egerlandstrasse 5 91058 Erlangen Germany; ^5^ Department of Trauma Hand, Plastic and Reconstructive Surgery University Hospital Würzburg Oberdürrbacher Str. 6 97080 Würzburg Germany; ^6^ Institute of Biomaterials Department of Materials Science and Engineering Friedrich‐Alexander University of Erlangen‐Nürnberg Cauerstr. 6 91058 Erlangen Germany; ^7^ Phil and Penny Knight Campus for Accelerating Scientific Impact University of Oregon 1505 Franklin Blvd Eugene OR 97403 USA

**Keywords:** 3D model systems, astrocytes, Ca^2+^‐Imaging, cortical neurons, hyaluronic acid, melt electrowriting

## Abstract

3D neuronal cultures attempt to better replicate the in vivo environment to study neurological/neurodegenerative diseases compared to 2D models. A challenge to establish 3D neuron culture models is the low elastic modulus (30–500 Pa) of the native brain. Here, an ultra‐soft matrix based on thiolated hyaluronic acid (HA‐SH) reinforced with a microfiber frame is formulated and used. Hyaluronic acid represents an essential component of the brain extracellular matrix (ECM). Box‐shaped frames with a microfiber spacing of 200 µm composed of 10‐layers of poly(ɛ‐caprolactone) (PCL) microfibers (9.7 ± 0.2 µm) made via melt electrowriting (MEW) are used to reinforce the HA‐SH matrix which has an elastic modulus of 95 Pa. The neuronal viability is low in pure HA‐SH matrix, however, when astrocytes are pre‐seeded below this reinforced construct, they significantly support neuronal survival, network formation quantified by neurite length, and neuronal firing shown by Ca^2+^ imaging. The astrocyte‐seeded HA‐SH matrix is able to match the neuronal viability to the level of Matrigel, a gold standard matrix for neuronal culture for over two decades. Thus, this 3D MEW frame reinforced HA‐SH composite with neurons and astrocytes constitutes a reliable and reproducible system to further study brain diseases.

## Graphical abstract

A reliable matrix formulation based on ultra‐soft thiolated hyaluronic acid (HA‐SH) reinforced by box‐shaped microfiber frames allows to setup 3D cell culture models suitable to investigate brain disease pathologies. Combined microfiber frames and HA‐SH prevent the HA‐SH from losing its form stability and drying out. Astrocytes represent essential physiological key players required for neuronal maturation and network firing activity.

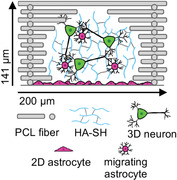

## 1 Introduction

The use of 3D in vitro cell culture models has increased rapidly in recent years for neuroscience research. Hydrogel matrices are used for brain organoid culture and provide a supportive environment for the self‐assembly of cells. Such 3D cultures require a cell‐compatible and, ideally, printable matrix that provides outcomes more reflective of the in vivo environment. As alternative approaches, human cortical spheroids^[^ ^1^ ^]^ or biomimetic 3D neuronal cultures using chitosan microbeads^[^ ^2^ ^]^ have been shown to result in functional neuronal network formations. Finding the ideal hydrogel for 3D cultures of cells of the central nervous system can be challenging due to the low elastic modulus of native brain tissue which ranges between 30–500 Pa.^[^ ^3^ ^]^ The mechanical properties of the brain differ greatly between embryogenesis and adulthood. In the developing brain, stiffness gradients as well as chemical cues guide axon growth.^[^ ^4^ ^]^ In addition, the brain extracellular matrix contains numerous biochemical components that vary between the basement membrane, the perineural net, and the neural interstitial matrix.^[^ ^5^ ^]^

Previous 3D neuronal in vitro cultures have demonstrated that neurons form more extensive networks in ultra‐soft matrices.^[^ ^6^ ^]^ However, such matrices that mimic native brain tissue are especially difficult to handle, which cause challenges for performing many experiments. The reinforcement of ultra‐soft matrices with 3D‐printed microfibers can solve this problem by providing overall stability to the matrix for handling while still maintaining its soft properties on the cellular level.

Previously, we described the reinforcement of Matrigel with poly(ɛ‐caprolactone) (PCL) microfiber frames produced via melt electrowriting (MEW).^[^ ^7^ ^]^ MEW is a high‐resolution additive manufacturing technology that deposits microscale fibers into complex structures, layer‐by‐layer.^[^ ^8^ ^]^ The PCL microfiber frame porosity is greater than 90%,^[^ ^9^ ^]^ but this is sufficient to improve handling and even increase the mechanical stability of matrices for the purpose of cartilage repair.^[^ ^10^ ^]^ Using this microfiber reinforcement principle, we previously created 3D models of transfected fibroblasts as well as cortical neurons in MEW‐frame‐reinforced Matrigel that allowed proper handling during structural investigations of the neuronal network formed and functional analysis using 3D patch‐clamp recordings.^[^ ^7^ ^]^ However, Matrigel possesses distinct disadvantages with batch‐to‐batch variations and its tumor origin which prevents translation into clinical settings.^[^ ^11^ ^]^ Nevertheless, Matrigel is widely recognized as the “gold standard” matrix within neuroscience for 3D cell culture. To allow clinical translation, defined and standardized 3D matrices of tissue‐relevant ECM components are urgently needed.

Here, we compared Matrigel with Glycosil, a commercially available thiolated HA for their performances on neuronal activity. HA was used due to its pronounced presence within the perineural net and neural interstitial matrix of the brain ECM. Furthermore, Glycosil was also compared to alginate, which has been used for 3D neuronal cultures,^[^ ^6^, ^12^ ^]^ and an oxidized form alginate dialdehyde combined with gelatin (ADA‐GEL).^[^ ^13^ ^]^ Moreover, we synthesized and formulated our own hyaluronic acid (HA) – a based matrix that was crosslinked with poly(ethylene glycol) diacrylate (PEGDA) with a high molecular weight. All hydrogels used were reinforced with melt electro‐written PCL microfibers.

## 2 Results and Discussion

To identify an ideal hydrogel for 3D cultures of cells of the central nervous system, challenges such as a low elastic modulus comparable to native brain tissue and a surrounding resembling brain ECM have to be taken into account. Cortical neurons have been successfully grown in Matrigel.^[^ ^7^, ^14^ ^]^ The origin of Matrigel envisions a cocktail of growth factors and ECM proteins secreted by a mouse sarcoma. Thus, for translational approaches, a reliable and defined matrix is essential to overcome the disadvantage of using a product with a tumor origin.^[^ ^11^ ^]^ HA represents one of the major components of brain ECM, specifically the perineuronal net and the neural interstitial matrix.^[^ ^15^ ^]^ Functionalized or natural HA and its blends have also been shown to be suitable for cellular growth in other 3D models, e.g., brain‐like tissue or cartilaginous constructs.^[^ ^5^, ^16^ ^]^

### 2.1 Cortical Neurons Show Low Viability in Glycosil Independent of Supplements Added

**Figure 1:**
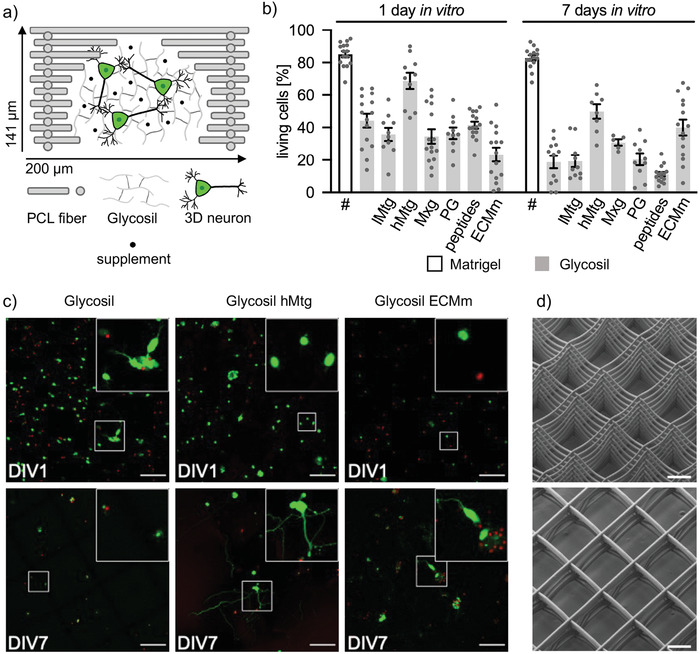
Glycosil supplementation has limited effects on neuronal cell viability. a) Schematic representation of a single 200 µm box of a PCL frame (grey bars) containing Glycosil (grey lines), 3D neurons (green), and supplement (black dots). b) Viability of cortical neurons in Glycosil containing different supplements (lMtg low Matrigel; hMtg high Matrigel; Mxg Maxgel; PG proteoglycan; peptides – RGD, IKVAV, YIGSR; ECMm mixture of various supplements) at DIV1 and 7, compared to viability in Matrigel (marked with #, data from a previous publication^[^ ^7b^ ^]^). A detailed description of the supplements and their concentrations used is found in Table 1. Cell viability was quantified by cell counting in five images per experiment. Each grey dot represents one analyzed image. Two or three independent experiments were performed (*n* = 2 − 3). Error bars represent SEM values. c) Representative images of selected data are shown in b. Living cells are shown green and dead cells in red. The upper right images represent higher magnifications of cells marked with white boxes. Scale bar: 100 µm. d) Scanning electron microscopy images of PCL‐frames with 200 µm hatch spacing. Upper image: top‐down view; lower image: bottom‐up view. Scale bar 100 µm.

HA is commercially available as Glycosil and was used to study the growth of murine cortical neurons. Therefore, mouse cortical neurons were isolated from embryonic stage E15‐17 and seeded into fiber‐reinforced Glycosil (**Figure** **1**a). Previously, we described how PCL frames were made via MEW.^[^ ^17^ ^]^ Reinforcing PCL MEW frames were fabricated with 10 layers and a hatch spacing of 200 µm using 9.7 ± 0.2 µm diameter fibers. The average thickness of the MEW frames was 141.4 ± 5.7 µm with spaces between PCL fiber layers that allow permeability by the hydrogel and even growth of neurites between single PCL layers. The PCL frames were cut as 9.8 mm discs that fit into 24‐well plates. The PCL‐frame (Figure 1d) allowed for easy handling and transfer of neuronal cultures. Neuronal cell viability in Glycosil was assessed at days in vitro (DIV) 1 and 7 and compared to Matrigel (DIV1: 85 ± 2%; DIV7: 83 ± 2%) (Figure 1b).^[^ ^7b^ ^]^ Viability in pure Glycosil was low at DIV1 (44 ± 4%) and even lower at DIV7 (19 ± 4%). To increase the cell viability of cortical neurons, several supplements were added to Glycosil (**Table** **1**). Supplementing Glycosil with 0.2 µg µl^−1^ of Maxgel (Mxg) which is a human non‐tissue specific ECM, containing collagens, laminin, fibronectin, tenascin, elastin, and a number of proteoglycans and glycosaminoglycans did not significantly improve cell viability compared to pure Glycosil.

**Table 1:** Supplements of Glycosil used to improve cell viability

Name	Supplement (order number)	Concentration (corresponding data)	Manufacturer
	Matrigel (734‐0271)	2.5 µg µL^–1^ (hMtg); 0.2 µg µL^–1^ (lMtg)	Corning, NY, USA
	MaxGel (E0282)	0.2 µg µL^–1^ (Mxg)	Sigma‐Aldrich, St. Louis, MO, USA
	Proteoglycan (P5864)	1 µg µL^–1^	Sigma‐Aldrich, St. Louis, MO, USA
ECM mixture (ECMm)	Tenascin (CC115)	2 ng µL^–1^	Sigma‐Aldrich, St. Louis, MO, USA
	Proteoglycan (P5864)	50 ng µL^–1^	Sigma‐Aldrich, St. Louis, MO, USA
	Fibronectin (F1056)	10 ng µL^–1^	Sigma‐Aldrich, St. Louis, MO, USA
	Laminin (LN111)	4 ng µL^–1^	Biolamina AB, Sundbyberg, Sweden
	Thiolated peptides (RGD, IKVAV, YIGSR)	150 ng µL^–1^, 1:1:1	AG Maric, Rudolf Virchow Center, Würzburg, Germany
Thiolated peptides (RGD, IKVAV, YIGSR)		150 ng µL^–1^, 1:1:1	AG Maric, Rudolf Virchow Center, Würzburg, Germany
NTF	BNDF, CNTF	10 ng mL^−1^	AG Sendtner, Institute for Clinical Neurobiology, Würzburg, Germany

Similarly, the addition of proteoglycans (PG) from bovine nasal septum containing a high degree of chondroitin sulfate, another component of the brain ECM, resulted in low cell viability at DIV14 (Figure 1b). Moreover, the supplementation of Glycosil with peptides targeting the integrin receptor, i.e., RGD, YIGSR, IKVAV of which the latter two sequences are derived from laminin, a glycoprotein of the brain extracellular matrix,^[^ ^17^ ^]^ had no noticeable effect. A mixture of various ECM proteins and peptides (ECMm) however could increase cell viability at DIV 7 by at least 40 ± 5%. Still, the most pronounced effect on cell viability was observed following the supplementation of Glycosil with Matrigel. The addition of 0.2 µg µl^−1^ of Matrigel (low concentration lMtg) to Glycosil had no large effect on cellular survival (DIV1: 36 ± 4%; DIV7: 19 ± 4%), the addition of 2.5 µg µl^−1^ Matrigel (high concentration hMtg) however increased cell viability (DIV1: 69 ± 5%; DIV7: 50 ± 4%). Glycosil supplemented with hMtg also allowed neuronal network formation (Figure 1c). In contrast, pure Glycosil was not sufficient to allow neuronal viability and maturation. The addition of single components not crosslinked to the matrix such as proteoglycans or peptides were unable to promote cortical networks.

### 2.2 Neuronal Viability in Glycosil Remains low in the Presence of Astrocytes

**Figure 2:**
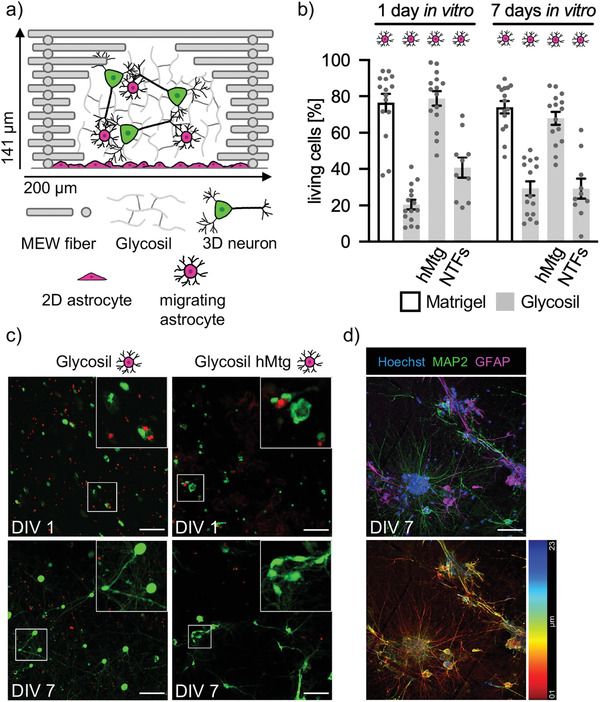
Neuronal viability in Glycosil remains low in the presence of 2D astrocytes. a) Schematic representation of a single 200 µm box of a PCL frame (grey bars) containing Glycosil (grey lines) and 3D neurons (green), as well as a 2D monolayer of astrocytes and migrating astrocytes (magenta). b) Viability of cortical neurons in Glycosil on an astrocyte monolayer and with or without addition of neurotrophic factors (NTFs; BDNF and CNTF) at DIV1 and 7, compared to viability in pure Matrigel (4.5 mg/ml) with astrocytes or Glycosil supplemented with Matrigel (hMtg). Cell viability was quantified by cell counting in five images per experiment. Grey dots represent the analyzed images. Magenta cell reflects the addition of 2D astrocytes. Two or three independent experiments were performed (*n* = 2 − 3). Error bar: SEM. c) Representative images of selected data are shown in b. Living cells are shown green and dead cells in red. White boxes mark the areas shown enlarged in the upper right of the original images. Scale bar:100 µm. d) Immunocytochemical staining of 3D cortical neurons (MAP2, green) and astrocytes (GFAP, magenta) in Glycosil‐PCL composites at DIV7 (nuclei/Hoechst, blue). Scale bar: 50 µm. The temporal color code projection below indicates the location of stained cells in the image stack, given in µm from red 1 µm to blue 23 µm.

Astrocytes play a critical physiological role in brain development and function by secretion of signal molecules, maintaining ion homeostasis, clearing of neurotransmitters, and by actively modulating neuronal activity through perisynaptic processes.^[^ ^18^ ^]^ Co‐cultures of neurons and astrocytes in vitro promote neuron survival and are regularly used in 2D cell culture models.^[^ ^19^ ^]^ In recent years, astrocytes have also been successfully used in models to improve neuron survival and neurite outgrowth.^[^ ^14^, ^20^ ^]^ Astrocytes grow well and maintain a high viability in reinforced Matrigel (Figure S1, Supporting Information). As supplementation of Glycosil could not generate the desired cell viability, a layer of 2D astrocytes was seeded below the PCL‐frame‐Glycosil composite (**Figure** **2**a). However, cell viability in Glycosil supported by astrocytes remained low (DIV1: 21 ± 3%; DIV7: 29 ± 4%) (Figure 2b). Interestingly, neurons formed a network at DIV7 (Figure 2c), which was not observed in Glycosil without astrocytic support (Figure 1c). Cortical neurons cultured in astrocyte supported Glycosil and further supplemented with 2.5 µg µl^−1^ Matrigel (hMtg) exhibited viability rates of 77 ± 5% at DIV1 and 74 ± 3% at DIV7 comparable to previous data in pure Matrigel. To investigate if neurotrophic factors (NTF) influence neuronal survival rates, brain‐derived neurotrophic factor (BDNF) and ciliary neurotrophic factor (CNTF) were added to the medium (NTFs, Table 1). However, no positive effect on cell viability could be observed (Figure 2b). It needs to be pointed out that in cell viability tests, no differentiation between living/dead neurons and astrocytes can be made. Immunocytochemical staining against the dendritic marker MAP2 and the astrocyte marker glial fibrillary acidic protein (GFAP) at DIV7 revealed that astrocytes migrated into Glycosil and captured close proximity to neurons, which formed a network with extensive dendrites (Figure 2d). Although astrocytes seemed to have a small promoting effect on neuronal survival resembling their physiological function during the development of the brain, Glycosil as a matrix did not enable a sufficient number of cortical neurons to survive as a prerequisite for functional network formation needed to study disease mechanisms of the brain.

### 2.3 Use of Further Matrices to Establish a 3D cell Culture Model with High Neuronal Viability During Maturation

**Figure 3:**
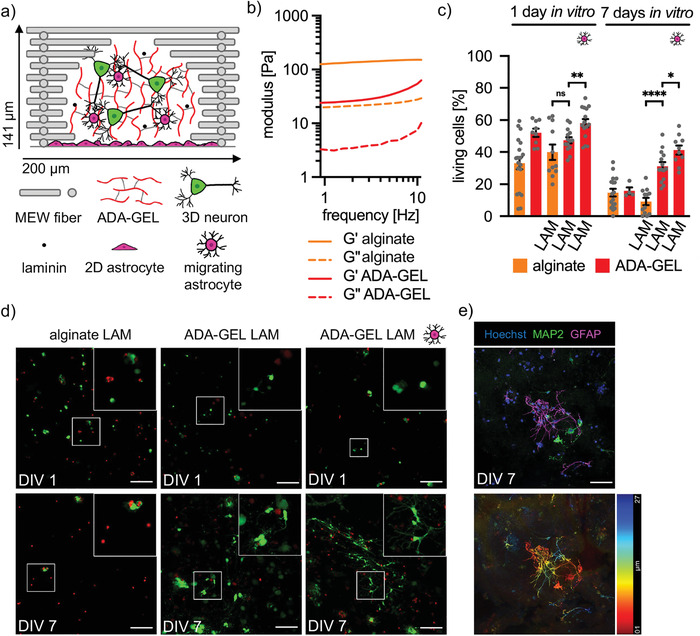
The presence of 2D astrocytes significantly increase neuronal viability in ADA‐GEL. a) Schematic representation of a single 200 µm box of a PCL frame (grey bars) containing ADA‐GEL (red lines), 3D neurons (green), laminin (black dots), as well as a 2D monolayer and migrating astrocytes (magenta). b) Rheological characterization of ADA‐GEL and alginate. Storage (G’) and loss moduli (G’’) were obtained from *n*  =  3 independent experiments. c) Viability of cortical neurons in alginate and ADA‐GEL with and without laminin supplementation or 2D astrocytes at DIV1 and 7. Cell viability was quantified by cell counting in five images per experiment. Each grey dot represents one analyzed image. Magenta cell reflects the addition of 2D astrocytes. Two or three independent experiments were performed (*n* = 2 − 3). Values of significance *p < 0.05, **p < 0.01, ****p < 0.0001, ns = not significant Error bar: SEM. d) Representative images of selected data are shown in c. Living cells are shown in green and dead cells in red. White boxes mark areas shown at higher magnification in the upper right of the images. Scale bar: 100 µm. e) Immunocytochemical staining of 3D cortical neurons (MAP2, green) and astrocytes (GFAP, magenta) in ADA‐GEL‐PCL composites at DIV7 (nuclei/Hoechst, blue). Scale bar: 50 µm. The temporal color code projection below shows the location of stained cells in the image stack, from red 1 µm to blue 27 µm.

As HA in the form of Glycosil proved not to be a viable alternative to Matrigel, cortical neurons were cultured in alginate as well as in its oxidized form ADA with crosslinked gelatin (ADA‐GEL) (**Figure** **3**a). Both alginate and ADA‐GEL have been described to serve as a suitable matrix for neuronal growth.^[^ ^6^, ^13^ ^]^ To estimate, if both matrices resemble stiffnesses comparable to brain tissue, rheological measurements of both matrices were performed without PCL frame. A frequency sweep was performed between 1 and 10 Hz at 0.1% strain and storage (G’) and loss moduli (G’’) were obtained (Figure 3b). At 5 Hz, ADA‐GEL (G‘: 33 Pa, G‘‘: 5 Pa) displayed lower moduli compared to alginate (G‘: 146 Pa, G‘‘: 23 Pa). Hence, the elastic modulus of ADA‐GEL was similar to soft Matrigel (4.5 mg mL^–1^), which has been successfully utilized for 3D cortical networks.^[^ ^7b^ ^]^

The viability of cortical neurons in alginate decreased rapidly (DIV1: 33 ± 4%; DIV7: 15 ± 2%), even when the ECM protein laminin (LAM) was supplemented (DIV1: 40 ± 5%; DIV7: 9 ± 3%) (Figure 3c). For laminin, the isoform LN111 was used which has been shown to increase cell attachment and neurite extension activities.^[^ ^22^ ^]^ Neurons in ADA‐GEL behaved similarly (DIV1: 52 ± 3%; DIV7: 16 ± 2%), but the addition of laminin could significantly increase viability at DIV7 (DIV1: 48 ± 2%; DIV7: 31 ± 3%, ****p < 0.00001). In previous studies, laminin has also been shown to improve neuroblastoma cell attachment to alginate,^[^ ^21^, ^22^ ^]^ while the addition of laminin crosslinked via amine groups to the aldehyde groups of ADA‐GEL enhanced the 3D neuron outgrowth of human‐induced pluripotent stem cell‐derived neurospheres.^[^ ^13b^ ^]^ In this context, a 2D layer of astrocytes could further significantly increase viability at both DIV1 (58 ± 3%, **p 0.00287) and DIV7 (41 ± 3%, *p 0.01766). Minor network formation was observed in ADA‐GEL LAM with and without astrocytes, but not in alginate LAM (Figure 3d). Staining revealed that migrated astrocytes outnumbered neurons in ADA‐GEL (Figure 3e). Although both, alginate and ADA‐GEL were used at a low concentration to exhibit similar elastic moduli as identified as beneficial to setup 3D neuronal models in Matrigel or Glycosil, neuronal maturation was low in alginate and ADA‐GEL. The low performance of alginate compared to ADA‐GEL might be represented by the increased stiffness of alginate compared to ADA‐GEL. The addition of laminin, an important adhesion molecule usually used as functionalizing material on surfaces to setup 2D or 3D neuronal cultures,^[^ ^23^ ^]^ helped to increase neuronal survival in ADA‐GEL but not in pure alginate. Taken together, a comparable stiffness to the endogenous stiffness of the brain is most probably important but apparently not the only prerequisite that allows the initiation of neuronal maturation.

### 2.4 HA‐SH – a Reliable and Reproducible Matrix Alternative

**Figure 4:**
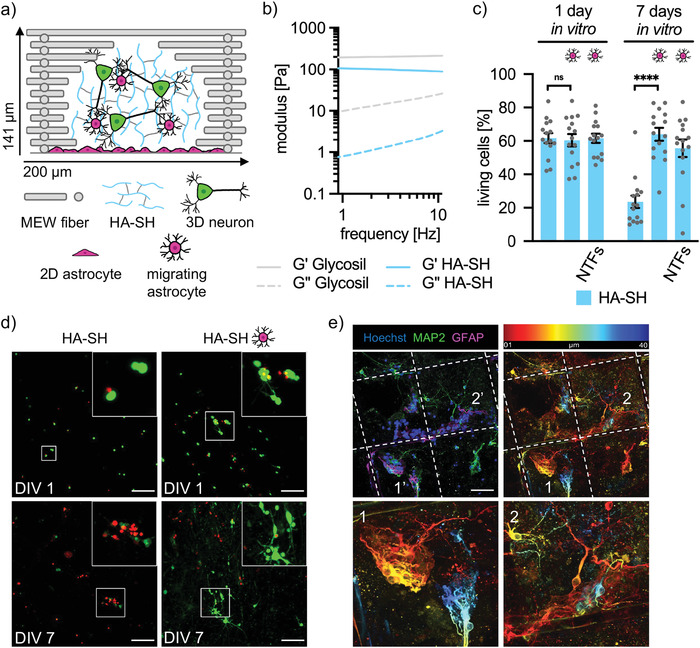
Astrocytes significantly promote neuronal viability in HA‐SH. a) Schematic representation of a single 200 µm box of a PCL frame (grey bars) containing HA‐SH (blue lines), 3D neurons (green), as well as a monolayer, and migrating astrocytes (magenta). b) Rheological characterization of HA‐SH compared to Glycosil. Storage (G’) and loss moduli (G’’) were obtained from *n*  =  3 independent experiments. c) Viability of cortical neurons in HA‐SH with and without 2D astrocytes or addition of neurotrophic factors (NTFs; BDNF and CNTF) at DIV1 and 7. Cell viability was quantified by cell counting in five images per experiment. Each grey dot represents one analyzed image. Magenta cells point to the addition of 2D astrocytes. Three independent experiments were performed (*n* = 3). Values of significance ****p < 0.0001, ns = not significant. Error bars: SEM. d) Representative images of selected data shown in c. Living cells are shown green and dead cells in red. White boxes mark the area enlarged in the upper right of presented images. Scale bar: 100 µm. e) Immunocytochemical staining of 3D cortical neurons (MAP2, green) and astrocytes (GFAP, magenta) in microfiber reinforced HASH composites at DIV7 (nuclei/Hoechst, blue). Scale bar: 50 µm. Temporal color code projection right demonstrates the location of stained cells in the image stack, from red 1 µm to blue 40 µm. Lower images show some marked areas with 1/1’ and 2/2’ in higher magnification. Note spreading of both cell types and astrocyte migration within various layers of the composite.

**Figure 5:**
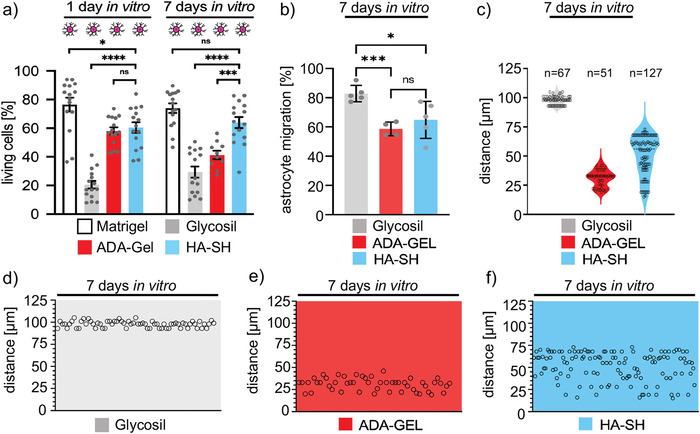
Cortical neurons in HA‐SH on a layer of 2D astrocytes exhibit high viability. a) Direct comparison of cell viability data in various types of ultra‐soft matrices, e.g. Matrigel (4.5 mg/ml), Glycosil, HA‐SH, and ADA‐GEL LAM (laminin added but no further ECM; cell viability data from Figures 2, 3, and 4). Note, that the neuronal viability in the presence of astrocytes is significantly higher for cells grown in HA‐SH compared to Glycosil and ADA‐GEL LAM and indistinguishable from Matrigel. Magenta cell indicates the addition of 2D astrocytes. b) Migration of astrocytes is shown as relative migration [%] into the hydrogels (Glycosil, ADA‐GEL, and HA‐SH). c) Violin plots showing astrocytes distribution along the respective hydrogels. Note, astrocytes migrate further into the Glycosil compared to HA‐SH and ADA‐GEL. Yet, a higher number of cells was present in HA‐SH (*n* = 127; compared to Glycosil *n* = 67; ADA‐GEL *n* = 51) and the distribution was more homogenous compared to the other hydrogels. d) Representation of the distance astrocytes migrated into Glycosil, e) ADA‐GEL, and f) HA‐SH. Values of significance *p < 0.05, ***p < 0.001 ****p < 0.0001, ns = not significant, Error bar: SEM.

Our viability analysis using various batches of the commercial Glycosil showed variability between independent experiments pointing to possibly lot to lot differences in oxidation degree and molecular weight. Therefore here, we used freshly thiolated and mostly linear HA crosslinked with PEGDA (HA‐SH) with a general HA chain length of 264 kDa within the matrix batch.^[^ ^16c^ ^]^ To investigate if linear polymer chains represent an advantage for the viability of cortical neurons, HA‐SH was analyzed in the same configuration with box‐shaped PCL frames and cortical neurons grown on top of an astrocytic feeding layer (**Figure** **4**a). Storage and loss moduli were obtained for Glycosil (at 5 Hz; G’: 206 Pa, G’’: 18 Pa) and HA‐SH (G″: 95 Pa, G″‘: 2 Pa), which even showed lower moduli than Glycosil (Figure 4b). Cortical neurons in fiber‐reinforced HA‐SH displayed a high initial viability at DIV1 (62 ± 3%) that dropped sharply at DIV7 (24 ± 4%), which was only slightly better than viability in Glycosil (Figure 4c). Furthermore, no network formation could be observed in HA‐SH (Figure 4d). However, a supporting layer of 2D astrocytes significantly increased cell viability at DIV7 (64 ± 4%, ****p < 0.00001) and enabled network formation (Figure 4c,d). The addition of NTF did not further promote viability. As seen before in Glycosil and ADA‐GEL, astrocytes did migrate from the 2D layer into HA‐SH towards neuronal proximity (Figure 4e). Vice versa, also neurons while forming their dendrites sense for astrocytic cell bodies (Supporting videos S1/S2). Astrocytes also seemed to accumulate near PCL fibers (dashed lines), probably due to local stiffness gradients. It is known that astrocytes change their morphology due to matrix stiffness. In soft substrates (150 Pa), astrocytes appear round and inactivated, however, they attach to stiff electrodes or PCL fibers and thus prefer stiffer environments compared to neurons.^[^ ^24^ ^]^ When directly comparing astrocyte‐supported and fiber‐reinforced matrices (data from Figures 2, 3, and 4; HA‐SH, ADA‐GEL LAM, and Glycosil ECM‐free), cell viability at DIV1 was highest in Matrigel, closely followed by HA‐SH and ADA‐GEL LAM. At DIV7, there was no significant difference in viability between Matrigel and HASH, which showed significantly better viability than ADA‐GEL LAM and Glycosil (**Figure** **5**a) arguing that the linear polymer chains of the provided thiolated HA and thereby softer hydrogels are generating a promoting effect for neuronal survival. Furthermore, the networks generated by the linear HA might also be more open as indicated by diffusion studies in similar hydrogels based on the same HA‐SH.^[^ ^16c^ ^]^ When investigating the relative migration of astrocytes into hydrogels Glycosil, ADA‐GEL, and HA‐SH, astrocytes migrated most in Glycosil (83 ± 5%), followed by HA‐SH (65 ± 12%) and ADA‐GEL (59 ± 4%) (Figure 5b). Similarly, the total distance moved was highest in Glycosil (98 ± 4 µm compared to HA‐SH 51 ± 16 µm, and ADA‐GEL 31 ± 7 µm; Figure 5c). These data follow the order of stiffness of the three hydrogels again supporting that astrocytes prefer stiffer hydrogels. However, looking at the distribution of migrated astrocytes, HA‐SH promoted a homogenous distribution of the migrated astrocytes, which was absent in Glycosil and only to some extent observed in ADA‐GEL (Figure 5d–f). Even though, the total number of astrocytes which have been detected in HA‐SH was higher compared to Glycosil and ADA‐GEL (Figure 5c). Therefore, the homogenous distribution of an increased number of migrating astrocytes in HA‐SH most likely accounts for the promoting effect on neuronal viability and maturation with the local supply of secreted growth factors in close neighborhoods to localized neurons within the hydrogel frame composite.

As a consequence, astrocyte‐supported HA‐SH was chosen as the best candidate for cortical cultures and used for all following experiments. Interestingly, there were no significant changes in viability of cortical neurons in Matrigel compared to Matrigel with astrocytes (Figure 1 compared to Figure 5) suggesting that the astrocytes have a supporting effect on neuronal survival with the secretion of growth factors and ECM proteins to pure hydrogels but no further support to Matrigel containing itself numerous growth factors and ECM proteins.

**Figure 6:**
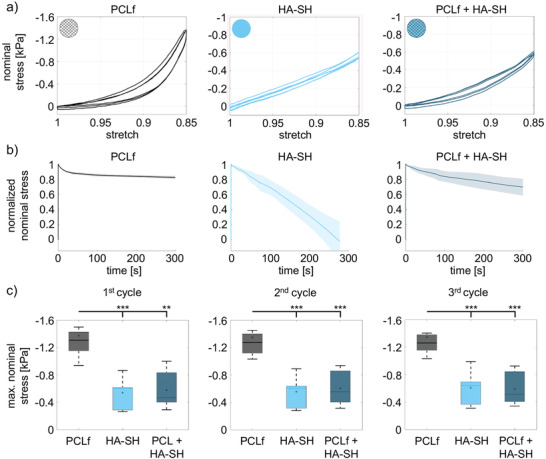
PCL frames prevent HA‐SH from losing stability. Large‐strain mechanical testing of PCL frames (PCLf), HA‐SH, and the combination of both, PCL frames and HA‐SH (PCLf+HA). a) Representative pre‐conditioning behavior in unconfined cyclic compression up to a maximum strain of 15% for PCL‐frame (*n* = 9), HA‐SH (*n* = 6), and PCL‐frame/HA‐SH samples (*n* = 6). b) Normalized stress relaxation behavior with corresponding standard deviations for PCL‐frame (*n* = 8), HA‐SH (*n* = 6), and PCL‐frame/HA‐SH samples (*n* = 6). c) Maximum nominal stress (mean ± SD) during the first, second, and third loading cycle for all samples tested in unconfined cyclic compression, significance value **p < 0.01, ***p < 0.001. Significances were calculated using students *t‐*test.

Rheological measurements showed ultra‐soft elastic moduli for HA‐SH, which are optimal for neuronal cells. However, these measurements were performed without the stabilizing PCL frame. Therefore, additional experiments were performed to measure the influence of the microfibers on the mechanical properties of HA‐SH. The average response of the PCL‐frame (adopted from,^[^ ^10b^ ^]^) pure HA‐SH and PCL‐frame/HA‐SH samples was analyzed during three cycles of unconfined compression with a maximum strain of 15% (**Figure** **6**a). All samples (PCL‐frame, HA‐SH, and PCL‐frame/HA‐SH) showed no clear difference between the three individual loading cycles (Table S1, Supporting Information). We note that this mechanical response with no notable softening upon preconditioning is different from the characteristic conditioning behavior of native brain tissue.^[^ ^26^ ^]^

The hysteresis – the area enclosed within the loading and unloading paths of the stress‐strain response – was most pronounced for the PCL‐frame and least pronounced for the pure HA‐SH. Therefore, while the pure HA‐SH is mostly elastic, combining it with the PCL‐frame adds certain viscous effects. To quantify the stress relaxation response, the samples were held at a constant deformation of 15% compression for 300 s (Figure 6b). Interestingly, the stress had completely relaxed after 270 s for the HA‐SH samples. Besides handling advantages, the combination of PCL‐frame and HA‐SH prevented the HA‐SH from losing its form stability and drying out. Correspondingly, the PCL‐frame/HA‐SH composites relaxed to 70% of the initial stress value only within 300 s. Figure 6c summarizes the maximum nominal stresses for pure PCL‐frame and HA‐SH as well as PCL‐frame/HA‐SH samples reached during cyclic loading (Figure 6a). For the first cycle, there were significant differences between the PCL‐frame and both the HA‐SH (***p  =  0.0007) as well as PCL‐frame/HA‐SH (**p  =  0.0014) maximum stress values (Table S1, Supporting Information). Interestingly, there was no significant difference in the maximum nominal stresses between the pure HA‐SH and the PCL‐frame/HA‐SH composites.

In summary, the PCL frames prevented the ultra‐soft matrix HA‐SH from losing its stability. Elasticity was, however, not significantly altered between HA‐SH and HA‐SH reinforced by PCL frames.

### 2.5 HA‐SH in the Presence of Astrocytes Allows Neuronal Maturation and Functional Neural Network Formation

**Figure 7:**
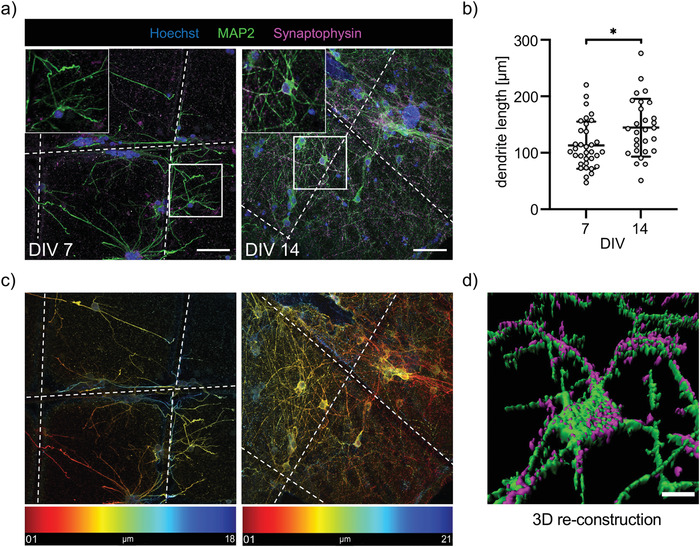
Cortical neurons in astrocyte‐supported HA‐SH form extensive networks. a) Immunocytochemical staining of 3D cortical neurons (MAP2, green) and synapses (synaptophysin, magenta) in astrocyte‐supported HASH‐PCL composites at DIV7 and 14 (nuclei/Hoechst, blue). Dashed white lines indicate the PCL fibers. Marked white boxes are represented at higher magnification in the upper left of the images. Scale bar: 50 µm. b) Comparison of dendrite length at DIV 7 and 14. Each grey dot represents one measured dendrite. Values of significance *p < 0.05. Error bars indicate standard deviation (SD). c) Temporal color code projection of the images exhibits the location of stained cells in the image stack, from red 1 µm to blue 18/21 µm. d) 3D reconstruction of the DIV14 network by neurons and astrocytes shows the boxed area in a.

**Figure 8:**
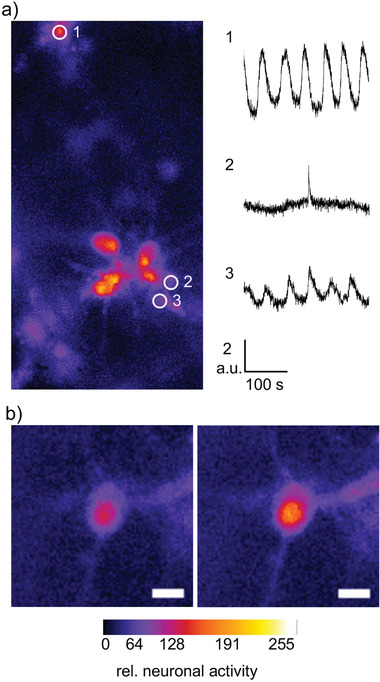
Cortical neurons grown in reinforced HA‐SH with astrocytes exhibit spontaneous neuronal network activity. a) Calcium imaging of cortical neurons in reinforced HA‐SH at DIV14. Three cells (1, 2, 3) were selected (left) and their spontaneous activity over 300 s (right) is shown. Traces 1 and 3 represent rather Ca^2+^‐oscillations most probably from astrocytes, trace 2 shows single action potential firing. b) Network activity of a single neuron. Change in relative neuronal activity over time is shown by color code from dark blue (no activity) to yellow (high activity). Scale bar refers to 10 µm.

Immunocytochemical stainings have demonstrated neuronal networks at DIV7 in astrocyte‐supported HA‐SH (Figure 4e). To resemble more detailed network development, staining against MAP2 which specifically stains components of the neuronal cytoskeleton and the synapse marker synaptophysin were performed at DIV7 and DIV14 (**Figure** **7**a). Signals are propagated from one neuron (presynaptic) to the next neuron (postsynaptic) via synaptic contacts. Neuronal networks increased in density between DIV7 and DIV14 with elevated numbers of dendrites and synapses. Quantification confirmed a significant increase in dendrite length (*p  =  0.0119) (Figure 7b) similar to previous data obtained from cortical neurons in 3D (Matrigel) compared to 2D.^[^ ^7b^ ^]^ Color‐code projections were used to visualize how cells spread over all layers of the culture and 3D reconstructions exhibit the localization of synaptophysin close to dendrites (Figure 7c,d). Hence, the ultra‐soft HA‐SH combined with an astrocytic layer followed by subsequent migration of astrocytes into the HA‐SH hydrogel to support neuronal maturation represents a controllable and reproducible matrix to study disease pathologies of the brain. Such investigations, however, require not only neuronal viability and structural marker expression which envision a mature neuronal network but also a functional representation of the network. As such, calcium imaging was performed to verify the function and maturation of 3D neuronal networks in fiber‐reinforced astrocyte‐supported HA‐SH (**Figure** **8**a,b). Spontaneous action potential firing was observed at DIV14 (Figure 8a, trace 2). The other recordings rather represent Ca^2+^ oscillations of unknown origin but might provide a hint for recording from the neighboring astrocytes (Figure 8a, traces 1 and 3). In conclusion, HA‐based matrices with stiffnesses comparable to brain tissue^[^ ^27^ ^]^ allow neuronal network formation in the presence of nutrient supply by neighboring astrocytes. Matrix stiffness is important for the viability of the neurons, but the linear polymer chain of the brain ECM component HA represents a reliable factor for neuronal survival. Astrocytes migrate and homogenously distribute into the gel due to its low stiffness and thus allow local ECM support for neighboring neurons. Within stiffnesses comparable to stages in brain development, astrocytes seem to function as pathfinders for neurons. Additionally, the migration into the matrix might also reflect the formation of tripartite synapses,^[^ ^28^ ^]^ as known from CNS tissue. Tripartite synapses are characterized by bidirectional communication between astrocytes and neurons. These specialized synapses are formed by pre‐ and postsynaptic neuronal cells enabling signal transduction together with neighboring astrocytes providing nutrient supply, clearing neurotransmitters from the synaptic cleft and recycling as well as facilitating neurotransmitters for further signal propagation.^[^ ^29^ ^]^

## 3 Conclusion

Using a bottom‐up approach, we have established a reliable and reproducible formulation using box‐shaped microfiber frames to stabilize ultra‐soft matrices used to enable the setup of a novel 3D model to study pathologies of brain diseases.

The model combines an ultra‐soft matrix of stiffness similar to that of brain tissue at the early stages of development with stiffer PCL frames that not only provide form stability of the matrix but also allow for easy handling during composite characterization.

Our main finding is that HA‐SH crosslinked with PEGDA constitutes a reproducible matrix that allows embryonic neurons to initiate maturation in the presence of neighboring astrocytes. Neuronal network formation was demonstrated at the structural level including sufficient synapse formation resulting in neuronal firing as a fundamental prerequisite of a mature network. Such a model offers now novel options to study disease mechanisms of rare neurological and neurodegenerative diseases.

## 4 Experimental Section

### MEW Processing

PCL frames were printed using a custom‐built MEW printer as previously described,^[^ ^7^, ^29^ ^]^ using medical‐grade PCL (PURASORB PC 12, Lot#1 712 002 224, 05/2018; Corbion Inc, Amsterdam, Netherlands) at 21 ± 2 °C and a humidity of 35 ± 10%. Parameters used with a laydown pattern of 0/90^o^ were 85 °C printer temperature, 3 bar of air pressure, 25G nozzle, and 6 kV voltage applied across a 4.5 mm collector distance.

### Synthesis of Thiolated Hyaluronic Acid (HA‐SH) and Polyethylene Glycol‐Diacrylate (PEGDA)

Hyaluronic acid sodium salt *M*_w_ = 1–2 MDa was purchased from Biosynth CarboSynth, Compton, UK. PEG Mn 6000 was purchased from Sigma Aldrich, St. Louis, MO, USA. In accordance with the protocol of Stichler et al., the synthesis of HASH was performed with a number of modifications, previously described.^[^ ^16^, ^30^ ^]^ PEGDA were synthesized according to Cruise et al.^[^ ^16^, ^31^ ^]^ The ready‐to‐use HA‐SH was obtained as white foam after freeze‐drying. The molecular weight of HA‐SH was *M_w_* = 264 kDa, DS 44 %.

### Alginate Di‐Aldehyde (ADA) Synthesis

As previously described by Sarker et al., alginate was partially oxidized by utilizing sodium (meta)periodate (NaIO_4_) as an oxidant.^[^ ^32^ ^]^ Since the procedure had to be adapted to the source of alginate, it will be briefly described. First, 10 g of sodium alginate from brown algae (VIVAPHARM alginate PH163S2, J. Rettenmaier & Söhne Pharma GmbH & Co. KG, Rosenberg, Germany) were dispersed in 50 mL of ethanol (99.8%). From here on, all further steps were performed under the exclusion of light. 1.337 g of NaIO_4_ (BioUltra, ≥ 99.5%, Sigma‐Aldrich Chemie GmbH) were dissolved in 50 mL ultrapure water (UPW, Direct‐Q, Merck Millipore) and slowly added to the stirring alginate dispersion. To quench the reaction after a total stirring time of 6 h at RT (22 °C), 10 mL of ethylene glycol (VWR Chemicals International) were added and stirred for another 30 min. After decanting the ethanol phase, ADA was dissolved in UPW and dialyzed against 15–17 L of UPW for 4 days. The dialysis tubes (SpectrumLabs) had a molecular weight cut‐off of 6–8  kDa and water was changed once a day. Finally, the ADA solution was frozen and lyophilized with an ALPHA 12 LDplus freeze‐dryer (CHRIST Gefriertrocknungsanlagen, Osterode am Harz, Germany).

### Mechanical Behavior

Rheological measurements were performed with a Physica MCR 301 rheometer (Anton Paar, Graz, Austria) in parallel plate configuration (25 mm diameter, 0.5 mm gap size). Matrices (500 µl) were placed on the rheometer at 37 °C. Amplitude sweeps were performed between 0.01% and 100% strain at 1 Hz. From this, linear viscoelastic regions were defined, and frequency sweeps were performed between 1 and 10 Hz at 0.1% strain (alginate, ADA‐GEL, Glycosil) or at 10% (HA‐SH). All experiments were performed in triplicates.

### Large‐Strain Mechanical Behavior

Mechanical characterization of PCL frame, HA‐SH, and PCL‐frame/HA‐SH composites under large strains was performed using a Discovery HR‐3 Rheometer (TA Instruments, Newcastle, USA). The sample diameter was identical to the PCL frame diameter with *d* =  9 mm for all samples. The samples were kept hydrated with a neurobasal medium during testing and it was assumed that they slide along the specimen holders″ surfaces, yielding a homogeneous deformation throughout the sample. To mimic in vivo conditions, all tests were performed at a constant temperature of 37 °C. The testing protocol started with a cyclic compression test with three loading cycles, a maximum strain of 15% and a velocity of 40 µm s^−1^. Subsequently, a stress relaxation test was conducted at the same maximum strain and velocity and a holding time of 300 s.

### Scanning Electron Microscopy

PCL frames were imaged using a Crossbeam 340 SEM equipped with a GEMINI e‐Beam column (Carl Zeiss Microscopy, Jena, Germany). Samples were sputter‐coated with a 4 nm platinum layer using an EM ACE600 sputter coater (Leica, Wetzlar, Germany).

### Isolation of Cortical Neurons

Primary cortical neuronal cultures were prepared at E17 from CD1 mouse embryos as described previously.^[^ ^7b^ ^]^ CD‐1 mice have been originally obtained from Charles River Sulzfeld, Germany, strain 022, and were further breed in the animal facility of the Institute for Clinical Neurobiology. Experiments were authorized by the local veterinary authority and Committee on the Ethics of Animal Experiments (Regierung von Unterfranken, license no.: FBVVL 568/200‐324/13).

### Isolation and Culture of Astrocytes

Primary astrocytes were isolated at P0‐P3 from CD1 mouse pups. Briefly, cortices were homogenized and put through a 70 µm cell strainer. Cells were counted, seeded in dishes, and cultured in DMEM supplemented with 10% fetal calf serum, 2 × 10^−3^ _M_ GlutaMAX, 1 × 10^−3^ _M_ sodium pyruvate, and 50 U mL^−1^ penicillin/streptomycin (Thermo Fisher Scientific, Waltham, United States) under standard growth conditions at 37 °C and 5% CO_2_. Cells were ready for experiments one week later after first splitting. Experiments were authorized by the local veterinary authority and Committee on the Ethics of Animal Experiments (Regierung von Unterfranken, license no.: FBVVL 568/200‐324/13).

### Matrix Preparation, Cell Seeding, and Culture

Two days before neuronal cell isolation, astrocytes were dissociated, counted and seeded in 2D monolayers. 20.000 astrocytes per ring were seeded into a 35 mm dish featuring 4 inner rings (627 170; Greiner Bio‐One, Kremsmünster, Austria). Prior to seeding of neurons, PCL frames were washed once with 70% ethanol, three times with ddH_2_O, once with PBS, and placed into the rings, with or without previously seeded astrocytes. After neuronal isolation, counting, and centrifugation, cell pellets were mixed with a precursor matrix solution (see matrix preparation), and 50–100 µL cell‐matrix mixture were pipetted onto each PCL frame. The final neuron concentration was 1000 cells µL^–1^ for cell viability analysis and 2000 cells µL^–1^ for all other experiments. After 20–30 min of incubation at 37 °C and 5% CO_2_, 3 mL Neurobasal medium containing 2 × 10^−3^ _M_ GlutaMAX and 2% (v/v) B27 supplement were added (Thermo Fisher Scientific, Waltham, MA, USA). BDNF and CNTF (10 ng mL^–1^) were included in the medium for some experiments. Cells were cultured under standard growth conditions at 37 °C and 5% CO_2_. Half of the medium was exchanged per week to retain growth factors secreted by neurons and astrocytes.

### Matrix Preparation—Matrigel

Matrigel was diluted with cell culture medium to a final concentration of 4.5 mg ml^–1^ (734‐0271; 9.1 mg ml^–1^; Corning, NY, USA).

### Matrix Preparation—Glycosil (HyStem)

0.5% w/v Glycosil, 0.5% w/v Extralink (HYS020‐1KT; Sigma‐Aldrich, St. Louis, MO, USA) were prepared in cell culture medium with supplements (see **Table** **1**). The various ECM components were pipetted into the hydrogel and by some Michael Addition chemical reactions bound to the Extralink reagent. However, there are limitations, e.g., proteoglycans cannot be bound by Extralink and were thus not linked to the hydrogel.

### Matrix Preparation—Alginate

Alginate was used as solution of 0.4% w/v alginate (PH176; J. Rettenmaier & Söhne Pharma, Rosenberg, Germany) with 1% v/v laminin (end concentration 1 ng µL^–1^; LN111; Biolamina AB, Sundbyberg, Sweden) in PBS. Crosslinking was done for 10 min with twice the volume of 100 m_M_ CaCl_2_ in cell culture medium.

### Matrix Preparation—ADA‐GEL

ADA‐GEL contained 0.4% w/v ADA (see *ADA synthesis*), 0.4% w/v gelatin (G2500; Sigma‐Aldrich, St. Louis, MO, USA), 1% v/v laminin (end concentration 1 ng µL^–1^; LN111; Biolamina AB, Sundbyberg, Sweden) in PBS. Crosslinking occurred for 10 min with twice the volume of 100 m_M_ CaCl_2_ in cell culture medium.

### Matrix Preparation—HA‐SH

HA‐SH was prepared using 0.75% w/v HA‐SH (264 kDa) in HEPES buffer (154 m_M_, pH 7.6), 0.75% PEGDA in PBS (see HA‐SH and PEGDA synthesis). The HA‐SH/PEGDA mixture was incubated for 25 min at 37 °C before addition of cells.

### Cell Viability

Viability of neurons and astrocytes was determined at DIV1 and 7. Cells were incubated for 30 min at 21 °C with 2 × 10^−6^ _M_ Calcein‐AM (green/living cells; Thermo Fisher Scientific, Waltham, MA, USA) and 2 × 10^−6^ _M_ ethidium homodimer I (red/dead cells; Sigma‐Aldrich, St. Louis, MO, USA) in PBS. The live/dead ratio was assessed by analyzing five image stacks per time point and experiment (*n* ≥ 2) using the Spots module of Imaris 7.7.2 (Oxford Instruments, Abingdon, UK).

### Immunocytochemical Staining

Neurons were stained for microtubule‐associated protein 2 (MAP2) and synaptophysin to assess neuronal network and synapse formation, while astrocytes were stained for glial fibrillary acidic protein (GFAP). All steps were performed at 21°C. Cells were fixed for 10 min with 2% paraformaldehyde and blocked/permeabilized for 30 min with 0.2% triton X‐100 and 5% goat serum in PBS. The primary antibodies anti‐MAP2, anti‐synaptophysin, or anti‐GFAP were applied for 1 h (all 1:500 in 5% goat serum in PBS; MAB3418, AB9272, AB5804; Merck Millipore, Burlington, MA, USA). After washing, cells were incubated with secondary Alexa488‐goat‐anti‐rabbit (GFAP), Cy3‐goat‐anti‐mouse (MAP2), or Cy5‐goat‐anti‐rabbit (synaptophysin) antibodies for 45 min (all 1:500 in 5% goat serum in PBS; Dianova, Hamburg, Germany). Samples were mounted on glass slides with Hoechst 33342‐containing ProLong Glass Antifade Mountant (Thermo Fisher Scientific, Waltham, MA, USA). Dendrite length was measured by using the FilamentTracer of Imaris (Oxford Instruments, Abingdon, UK) on the longest and fully traceable dendrites in each image.

### Astrocyte Migration

Image stacks for cortical neurons grown in hydrogel reinforced with PCL frames on top of a layer of astrocytes were taken. The distances of astrocyte migration into the hydrogel were determined by the location of the cell body within the image stacks. Distance between layers was 1 µm (total number of layers *n* = 77 − 81 for HA‐SH; *n* = 113 − 125 for Glycosil, *n* = 50 − 55 for ADA‐GEL). To allow comparison, the migrated distance of all cells was averaged and then divided by the total distance of each condition (Glycosil, ADA‐GEL, or HA‐SH). These values reflect the depth astrocytes migrated into the hydrogels (astrocyte migration [%].

### Confocal Microscopy and Image Acquisition

An Olympus IX81 microscope equipped with a FV1000 confocal laser scanning system, a FVD10 SPD spectral detector, and diode lasers of 405, 495, 550, and 635 nm (Olympus, Tokyo, Japan) was used for image acquisition of cell viability and immunocytochemistry experiments. Olympus UPLSAPO 20X (air, numerical aperture 0.75) and UPLFLN 40X (oil, numerical aperture 1.3) objectives were used. Maximum intensity projections, dynamic range adjustments, and temporal color coding of image stacks were done using ImageJ/Fiji 1.53 g.^[^ ^33^ ^]^ Imaris was used for 3D reconstruction (Oxford Instruments, Abingdon, UK).

### Live Imaging

Live‐cell imaging was performed with a 24‐channel incubator microscope (zenCELL owl; innoME GmbH, Espelkamp, Germany) at 37 °C and 5% CO_2_. Cells and HA‐SH were prepared and seeded as previously described in a 24‐well plate and subsequently imaged every 5 min for 48 h. A digital phase contrast was applied using the zenCELL owl software 3.2.

### Calcium Imaging

Neuronal activity was assessed using the calcium imaging technique. Cortical neurons were labeled for 15 min at 37 °C and 5% CO_2_ with 5 µ_M_ calcium indicator Oregon Green 488 BAPTA‐1AM (5 m_M_ stock in 20% Pluronic F127/DMSO; Thermo Fischer Scientific, Waltham, MA, USA). The imaging solution contained (in ×10^–3^ _M_) 119 NaCl, 4.5 KCl, 1 MgCl_2_, 2 CaCl_2_, 1.2 NaH_2_PO_4_, 26 NaHCO_3_, 10 glucose, 10 HEPES, pH 7.4, adjusted with NaOH. StreamPix4 software (Norpix, Montreal, Canada) was used to capture image series at 10 Hz with a Rolera XR Mono fast 1394 CCD camera (Qimaging, Surrey, Canada) and a cooled epifluorescent light source for 470 nm (Visitron Systems, Puchheim, Germany). Obtained data were analyzed using ImageJ.

### Statistical Analysis

Mean values, standard deviation, standard error of the mean (SEM), and statistical significance were calculated using GraphPad Prism 9.0.0 (Graphpad Software, San Diego, CA, USA). Statistical significance was estimated using an unpaired *t*‐test with Welch's correction with *p < 0.05, **p < 0.01, ***p < 0.001, ****p < 0.0001. Data were not assessed for normality, no test for outliers was conducted, and no sample size calculation was done.

## Conflict of Interest

The authors declare no conflict of interest.

## Author Contributions

C.V., P.D.D., T.B., S.B., J.T., R.D., and A.B. participated in research design. D.J., E.B., L.F., J.H., J.H., J.F., A.P., and M.A.M conducted experiments. D.J., E.B., J.F., and M.A.M. performed data analysis. C.V. and D.J. Wrote the manuscript with the help of all co‐authors.

## Supporting information

Supporting Information

Supplemental Video 1

Supplemental Video 2

## Data Availability

The data that support the findings of this study are available from the corresponding author upon reasonable request.
