# Supplementary material for: Reinforced Hyaluronic Acid‐Based Matrices Promote 3D Neuronal Network Formation
Source: Adv Healthc Mater. 2022 Sep 1;11(21):2201826. doi: 10.1002/adhm.202201826 (PMC11468248; doi:10.1002/adhm.202201826)
Supplement: Supplementary file 1 — Supporting Information [file ADHM-11-2201826-s001.pdf]

Supporting Information

Reinforced Hyaluronic acid-based Matrices promote 3D Neuronal Network Formation

*Dieter Janzen, Ezgi Bakirci, Jessica Faber, Julia Hauptstein, Arindam Pal, Leonard Forster, Jonas Hazur, Aldo R. Boccaccini, Rainer Detsch, Jörg Teßmar, Silvia Budday, Torsten Blunk, Paul D. Dalton, Carmen Villmann**

* Corresponding author

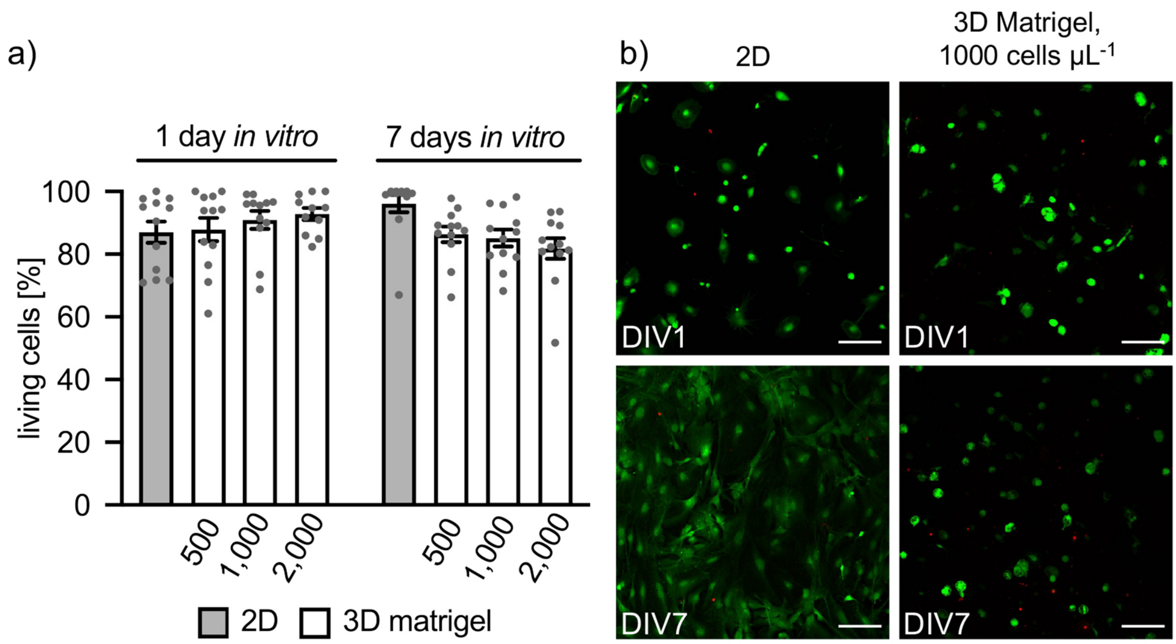

Figure S1. Viability of astrocytes in 2D and 3D Matrigel at DIV1 and 7. a) Astrocytes were seeded on glass cover slips (150000 cells per 35 mm dish containing 4 cover slips) or in reinforced Matrigel (4.5 mg mL^{-1}; 500, 1000, or 2000 cells μL^{-1}). Cell viability was quantified by cell counting in five images per experiment. Each grey dot represents one analyzed image. Three independent experiments were performed (n = 3). Living cells: grey bar 2D, white bars 3D. Error bar: SEM. b) Representative images of data shown in a. Living cells are shown green, dead cells in red. Scale bar: 100 µm.

Video S1. Live imaging of 3D cortical neurons and 2D astrocytes in HA-SH over 48 hours - 1

Video S2. Live imaging of 3D cortical neurons and 2D astrocytes in HA-SH over 48 hours - 2

Cortical neurons in fiber-reinforced HA-SH were seeded on top of 2D astrocytes and imaged for 48 hours using a zenCELL owl incubator microscope. Note neurite growth (white arrowheads) and neuron-astrocyte interaction (black arrowheads).

Table S1: Cyclic compression tests of PCL-frame, HA-SH, and PCL-frame/HA-SH

			PCL-frame	HA-SH	PCL-frame + HA-SH
		n	9	6	6
Maximum values of cyclic compression tests	1st cycle		-1.3730 ± 0.086	-0.5406 ± 0.2272	-0.5780 ± 0.2780
	2nd cycle		-1.3492 ± 0.064	-0.5572 ± 0.2270	-0.6064 ± 0.2533
	3rd cycle		-1.3484 ± 0.044	-0.6076 ± 0.2475	-0.5913 ± 0.2443
1st cycle vs 2nd cycle		p-value	0.8929 ns	0.9016 ns	0.8568 ns
1st cycle vs 3rd cycle		p-value	0.9284 ns	0.6356 ns	0.9316 ns
x vs PCL-frame	1st cycle	p-value	-	0.0007 ***	0.0014 **
	2nd cycle	p-value	-	0.0005 ***	0.0009 ***
	3rd cycle	p-value	-	0.0007 ***	0.0006 ***

Significance values: **p<0.01, ***p<0.001, ns = not significant, n = number of measured constructs.
